# Comparison of transposition and interposition methods in microvascular decompression for hemifacial spasm: an analysis of 109 cases performed by a single surgeon in a single-center retrospective study

**DOI:** 10.1007/s00701-024-06111-0

**Published:** 2024-05-14

**Authors:** Etsuko Owashi, Kazufumi Ohmura, Kenji Shoda, Tetsuya Yamada, Kiyomitsu Kano, Noriyuki Nakayama, Toru Iwama

**Affiliations:** Department of Neurosurgery, Gifu University Graduate School of Medicine, Gifu, Japan

**Keywords:** Hemifacial spasm, Microvascular decompression, Interposition, Transposition

## Abstract

**Background:**

Microvascular decompression (MVD), the standard surgical approach for hemifacial spasm (HFS), can be divided into the interposition and transposition methods. Although the risk of HFS recurrence following interposition has been reported, there is limited data comparing long-term outcomes between both methods performed by a single surgeon. This study aimed to investigate the efficacy of MVD techniques on HFS by comparing surgical outcomes performed by a single surgeon in a single-center setting.

**Methods:**

A total of 109 patients who underwent MVD were analyzed and divided into the transposition (86 patients) and interposition (23 patients) groups. Postoperative outcomes at 1 month and 1 year were assessed and compared, including rates of spasm relief, complications, and recurrence.

**Results:**

Outcome assessment revealed higher rates of early spasm relief in the interposition group (66.3% *vs.* 100%, transposition *vs.* interposition, respectively, *p* = 0.0004), although spasm relief at 1-year postoperatively was comparable between the two groups (84.9% *vs.* 95.7%, transposition *vs.* interposition, respectively, *p* = 0.2929). No significant differences were observed in complication and recurrence rates. Kaplan–Meier analysis demonstrated no significant differences in the duration of spasm resolution by MVD method (*p* = 0.4347, log-rank test).

**Conclusion:**

This study shows that both the transposition (Surgicel® and fibrin glue) and interposition (sponge) methods were excellent surgical techniques. The interposition method may achieve earlier spasm resolution compared to the transposition method.

## Introduction

Microvascular decompression (MVD) has emerged as the gold standard surgical treatment for hemifacial spasm (HFS) since its introduction in the 1970s owing to its safety and efficacy [6, 16]. This minimally invasive procedure can be performed based on various techniques broadly categorized as interposition and transposition methods [2]. The interposition method utilizes a soft material, often a small sponge or Teflon felt, inserted between the compressing blood vessel and the facial nerve at the root exit zone (REZ). This creates a barrier that prevents the vessel from compressing or irritating the nerve, effectively achieving facial nerve decompression. Pioneered by Jannetta et al. [6], this method remains the traditional and standard surgical approach worldwide. Meanwhile, the transposition method is an alternative approach, wherein the compressing blood vessel is moved away from its original position, transposing the vessel to a location where it no longer compresses the nerve. While both methods demonstrate favorable treatment outcomes in HFS, studies suggest a higher risk of facial spasm recurrence with the interposition method [17, 19].

Few studies have reported on the long-term outcomes of MVD for HFS performed by a single surgeon using standardized techniques [3, 9]. As such, it is important to consider that variations in surgical techniques among surgeons can affect comparisons in MVD treatment outcomes [1, 2]. In our institution, a single surgeon performs MVD for HFS, employing either the transposition method with sliced oxycellulose (Surgicel® FibrillarTM; Ethicon, Bridgewater, NJ, USA) and fibrin glue or the interposition method with a sponge. Leveraging this opportunity, this study aimed to investigate the treatment outcomes of MVD for HFS performed by a single surgeon using these standardized techniques (interposition *vs.* transposition) and elucidate the relationship between these surgical methods and treatment outcomes.

## Methods and materials

### Patient selection

**Fig. 1:**
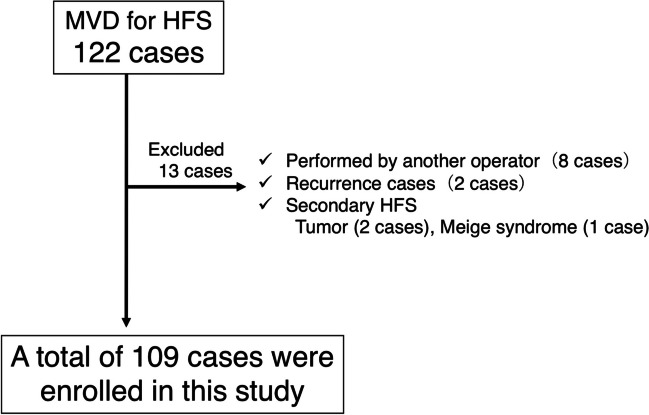
Summary of study inclusion criteria. n, number; MVD, microvascular decompression; HFS, hemifacial spasm

This retrospective study included 109 out of 122 MVD procedures performed at our institution between June 1, 2004, and March 31, 2021. Thirteen cases were excluded due to involvement of different surgeons, postoperative recurrence at other hospitals, and secondary HFS (Fig. 1). Medical records were reviewed to collect data, and additional surveys were conducted via telephone or questionnaire for patients with incomplete follow-up data. The patients were categorized into the transposition (*n* = 86) and interposition (*n* = 23) groups based on the surgical method. Written informed consent was obtained from all patients. This study was approved by the ethics committee of Gifu University Graduate School of Medicine (Number:2022–096).

### Surgical procedures

**Fig. 2:**
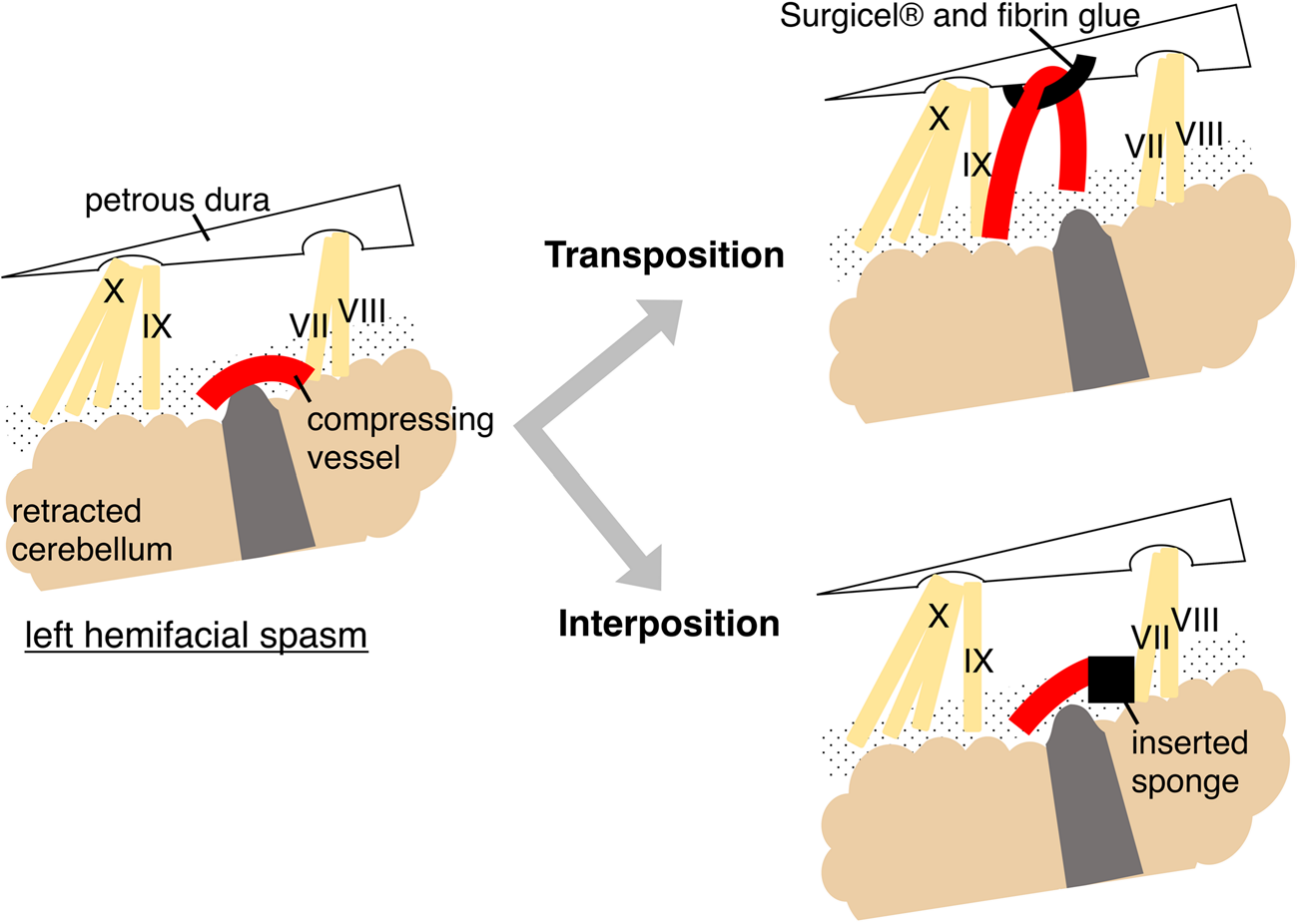
Illustrative presentation of both MVD methods. In microvascular decompression for hemifacial spasm, two different surgical procedures are employed. Transposition: the compressing vessel is moved away from the facial nerve. The vessel is then secured to the dural surface using sliced oxycellulose (Surgicel® FibrillarTM) and fibrin glue. Interposition: a sponge is interposed between the root exit zone and artery. The sponge is then secured with fibrin glue. VII, facial nerve; VIII, vestibulocochlear nerve; IX, glossopharyngeal nerve; X, vagus nerve

All 109 cases in this study were performed by a single surgeon (T. I.). Patients were placed in the park bench position under general anesthesia, and a 2.5 cm × 2.5 cm suboccipital craniotomy was performed. After opening the dura mater, sufficient cerebrospinal fluid was drained from the cisterna magna under microscopic visualization. The cerebellum was meticulously inspected, and the facial nerve and REZ were identified after slight elevation of the flocculus. Upon confirmation of vessel-nerve conflict, microsurgical dissection was carefully performed to free the vessel and the nerve from surrounding arachnoid adhesions, allowing optimal mobilization and decompression. Monitoring of abnormal muscle response (AMR), which was utilized in cases after January 2012, showed resolution in all cases. The decision of interposition or transposition method was not randomized. The surgeon first attempted transposition method on the compressed vessel. If the contact between the nerve and offending vessel could not be removed, the interposition method was employed. Figure 2 shows a schematic representation of both MVD procedures.

### Interposition method

Following mobilization and detachment of the compressing vessel from the nerve, a sponge was interposed between the REZ and artery. The sponge was then secured with fibrin glue. Decompression was confirmed before performing watertight closure. Muscle pieces were used, if necessary, to seal openings.

### Transposition method

**Fig. 3:**
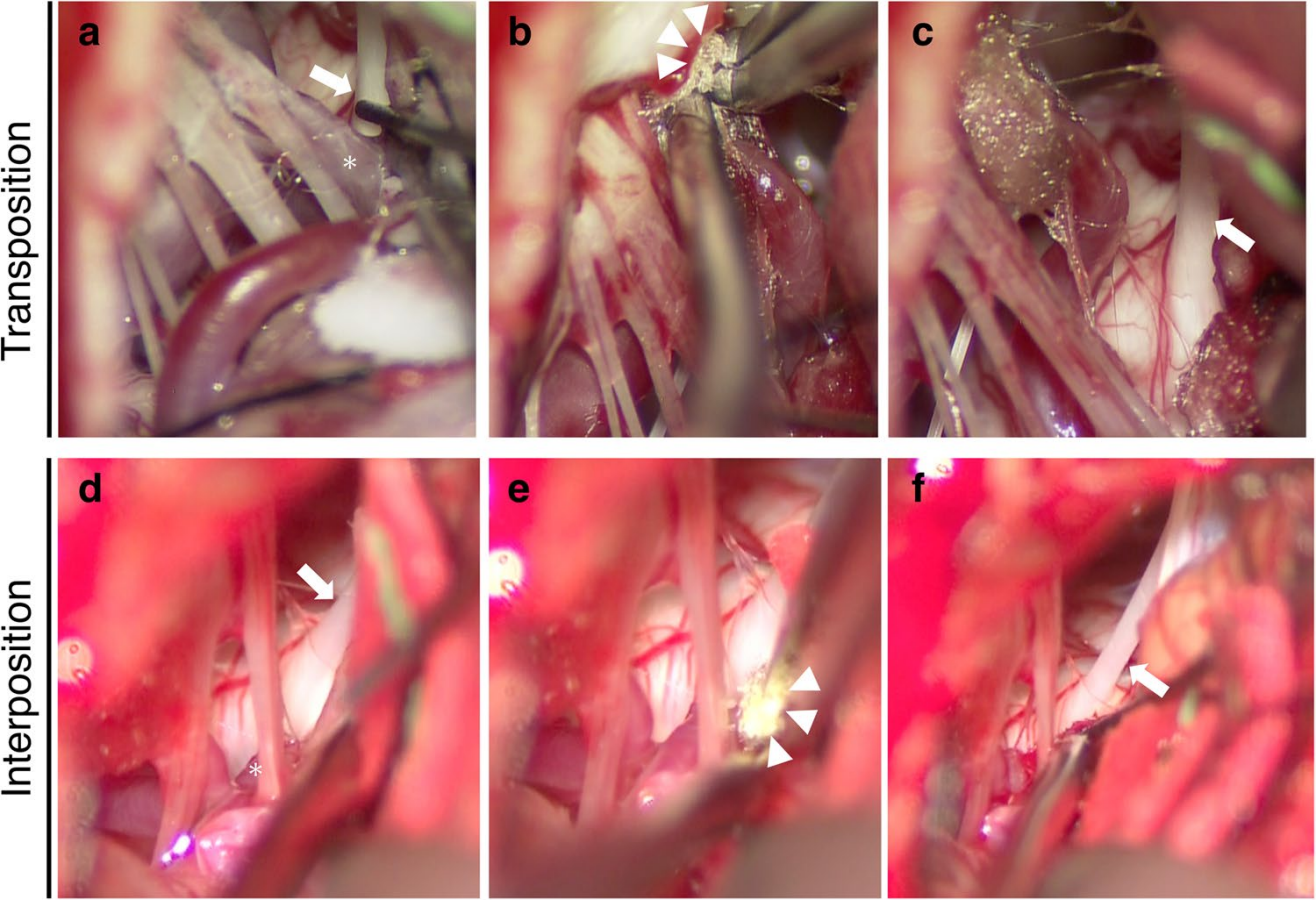
Representative intraoperative findings of both MVD groups. **a**–**c** Transposition method: **a** The asterisk indicates the posterior inferior cerebellar artery (PICA) compressing the facial nerve at the root exit zone (REZ) (arrow). **b** Surgicel® (arrowheads) and fibrin glue were used to secure the vessel to the dural surface. **c** The PICA is lifted and moved from the nerve (arrow). **d**–**f** Interposition method: **d** The asterisk indicates the PICA compressing the facial nerve at the REZ (arrow). **e** Arrowheads show the sponge inserted between the PICA and REZ. **f** The arrow indicates the left facial nerve. MVD, microvascular decompression

Following complete detachment of adherent facial nerve membranes, the compressing vessel was moved away from the REZ. The vessel was then secured to the dural surface using sliced oxycellulose (Surgicel® FibrillarTM) and fibrin glue. The remainder of the procedure followed the protocol described for the interposition method. Figure 3 shows representative intraoperative findings in both MVD groups.

### Outcome assessment

Treatment outcomes were assessed at 1 month and 1 year postoperatively. Spasm relief was categorized as excellent (complete resolution), good (partial resolution with patient satisfaction), or poor (worsening symptoms with patient dissatisfaction). Transient neurological symptoms were also documented. Recurrence was defined as reappearance of spasms or worsening of seizure frequency 1 year postoperatively [15].

### Statistical analysis

Continuous and categorical variables were assessed using the Wilcoxon rank test and Fisher's exact test, respectively. Kaplan–Meier analysis was utilized to compare spasm-free intervals. All statistical analyses were conducted using JMP 14.2 software, and statistical significance was set at a *p*-value < 0.05.

## Results

### Patient characteristics

Table 1 summarizes the patient backgrounds. The mean age was 58 years (range: 22–79 years), and 14.7% of the patients had a history of Botox treatment (botulinum toxin type A). Regarding HFS laterality, the interposition group demonstrated a higher prevalence of left-sided facial spasms (18/23, 78.3%) compared to the transposition group (54/86, 52.3%) (*p* < 0.032). No significant differences in age, sex, symptom duration, history of hypertension, and prior Botox treatment were observed between the two groups. The anterior inferior cerebellar artery (AICA) was the most commonly implicated vessel in HFS, followed by the posterior inferior cerebellar artery (PICA) and vertebral artery (VA). No significant differences in compressing vessels were noted between the two groups.

**Table 1:** Baseline characteristics of both MVD groups

Variable	All (*n* = 109)	Transportation (*n* = 86)	Interpostion (*n* = 23)	*P* value
Age, year (median, range)	58, 22–79	58, 22–79	68, 29–74	0.603
Gender (male, %)	41, 37.6	32, 37.2	9, 39.1	1
Location (Left, %)	63, 57.8	45, 52.3	18, 78.3	0.032^*^
Symptom duration, month (median, range)	36, 2–360	36, 3–360	48, 2–228	0.5358
Hypertension (n, %)	10, 9.3	6, 7.1	4, 17.4	0.2153
Botox treatment (n, %)	16, 14.7	13, 15.1	3, 13	1
Main compressing vessel (n, %)				
anterior inferior cerebellar artery	57, 52.3	45, 52.3	12, 52.2	1
posterior inferior cerebellar artery	52, 47.71	41, 47.67	11, 47.71	1
vertebral artery	16, 14.68	12, 14	4, 17.4	0.7416
multiple arteries	15, 13.8	11, 12.8	4, 17.4	0.5165

*n* number, *MVD* microvascular decompression. Asterisk indicates statistical significance

### Interposition method leads to early spasm relief but similar long-term outcomes

**Fig. 4:**
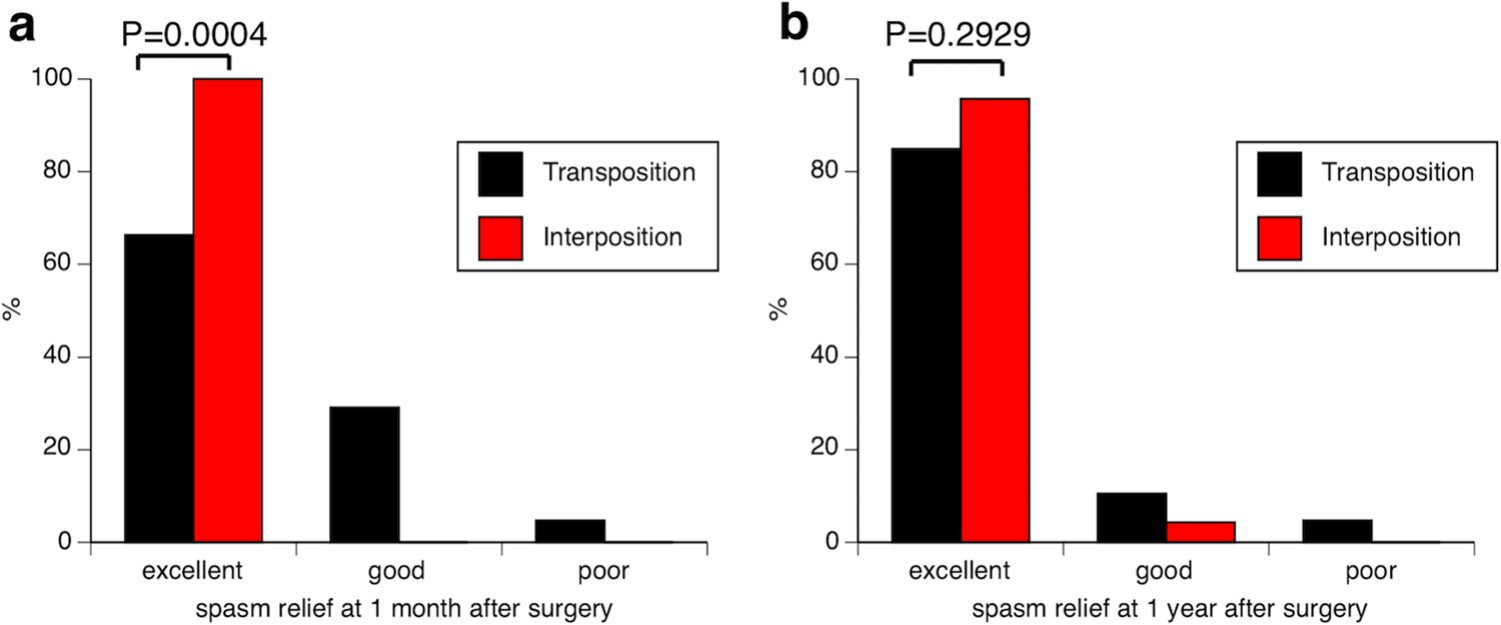
Comparison of spasm relief outcomes between both MVD groups. The bar graph shows outcomes measured 1 month (**a**) and 1 year (**b**) postoperatively. “Excellent” indicates complete spasm resolution, “good” indicates residual spasm with patient satisfaction, and “poor” indicates worsening spasm with patient dissatisfaction. At 1 month postoperatively, the interposition group shows a significantly higher rate of spasm resolution compared to the transposition group (*p* = 0.0004). However, spasm resolution is comparable between the two methods at 1-year postoperatively (*p* = 0.2929). MVD, microvascular decompression

To evaluate the outcomes of both methods, spasm relief at 1 month and 1 year postoperatively was compared. At 1 month postoperatively, the transposition group achieved excellent relief in 57 patients (66.3%), good relief in 25 (29.1%), and poor relief in 4 (4.7%). Meanwhile, the interposition group achieved excellent relief in all cases (100%), with no patients achieving good or poor relief. This showed that the interposition group displayed a significantly higher prevalence of excellent spasm relief (66.3% *vs.* 100%, transposition *vs.* interposition, respectively, *p* < 0.0004). At 1 year postoperatively, the transposition group achieved excellent relief in 73 patients (84.9%), good relief in 9 (10.5%), and poor relief in 4 (4.7%). In contrast, interposition group achieved excellent relief in 22 patients (95.7%) and good relief in 1 (4.3%), without any cases of poor relief. Excellent spasm relief rates at this time were comparable between the two groups (84.9% *vs.* 95.7%, transposition *vs.* interposition, respectively, *p* < 0.2929) (Fig. 4). Although none of the patients in the transposition group achieved excellent spasm relief within 1 month, 16 patients showed improvements in achieving excellent relief at 1 year postoperatively.

### Similar complications and recurrences rates between both groups

**Fig. 5:**
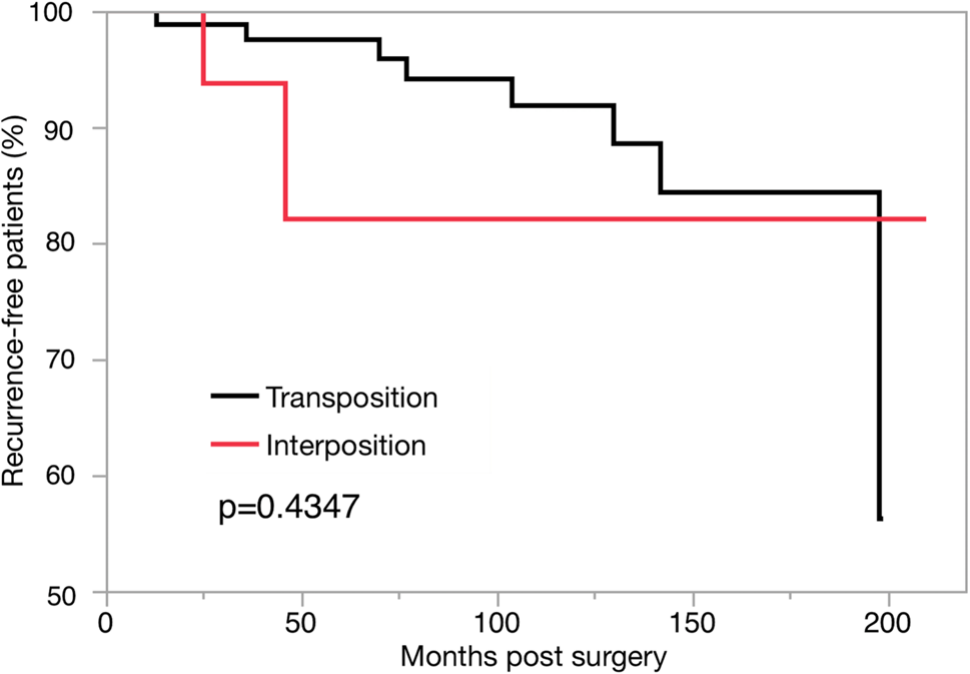
Spasm recurrence-free interval curves in both MVD groups. Kaplan–Meier analysis shows the recurrence-free interval in both techniques postoperatively (*p* = 0.4347, log-rank test). MVD, microvascular decompression

Complications and recurrence rates were also examined and compared. No mortalities were reported in neither groups. Complications in the transposition group included hearing loss (two cases), facial paralysis (two cases), and hoarseness (one case). However, only one case of hoarseness was reported in the interposition group. No significant differences in complications were observed between the two groups (5.8% *vs.* 4.3%, transposition *vs.* interposition, respectively, *p* = 1). Similarly, recurrence rates were comparable between the two, with eight and two cases in the transposition and interposition groups, respectively (9.3% *vs.* 8.7%, transposition *vs.* interposition, *p* = 1). In contrast, the follow-up duration significantly differed between the groups (40 months *vs.* 98.5 months, interposition *vs.* transposition, respectively, *p* < 0.0001) (Table 2). Kaplan–Meier analysis also revealed no significant differences in spasm-free intervals based on surgical method (*p* = 0.4347, Fig. 5). In subgroup analysis, 89 patients who had at least 3 years of follow-up were evaluated (transposition: 76 cases, interposition: 13 cases). Recurrence rates were comparable between the two groups, with seven and one cases in the transposition and interposition groups, respectively (9.2% *vs.* 7.7%, transposition *vs.* interposition, *p* = 1). Kaplan–Meier analysis also revealed no significant differences in spasm-free intervals based on the surgical method (*p* = 0.8586).

**Table 2:** Complication and recurrence of both MVD groups

Variable	All (*n* = 109)	Transposition (*n* = 86)	Interpostion (*n* = 23)	*P* value
Complication (n, %)	6, 5.5	5, 5.8	1, 4.3	1
Hoarseness	2, 1.8	1, 1.2	1, 4.3	NA
Hearing loss	2, 1.8	2, 2.3	0	NA
Facial paralysis	2, 1.8	2, 2.3	0	NA
Recurrence (n, %)	10, 9.2	8, 9.3	2, 8.7	1
Follow-up duration, month (median, range)	78, 12–210	98.5, 12–199	40, 12–210	< 0.0001^*^

Values for outcome are expressed as the number of patients (%), and values for follow-up are expressed as the median number of months (range). NA denotes not applicable. Asterisk indicates statistical significance

## Discussion

This study investigated the efficacy of the interposition and transposition methods for HFS performed by a single surgeon using standardized techniques. The interposition group demonstrated a significantly higher rate of complete spasm relief at 1 month postoperatively. However, rates of complete spasm relief and spasm-free intervals were comparable between the two groups at 1 year postoperatively.

Previous studies have reported complete spasm relief rates of approximately 85–90% following MVD for HFS, which is similar to those shown in the present study [5, 17]. Interestingly, symptom improvement was observed in 16 patients in the interposition group at 1 year postoperatively. This is consistent with reports on delayed spasm improvement several months after MVD [4, 8]. Our findings also revealed a significant difference in symptom improvement based on surgical technique. Namely, early spasm relief was more likely to occur following MVD using the interposition method. The reason why the interposition method results in early spasm relief is unclear. The insertion of the interposition prosthesis is feared to cause direct nerve damage [13]. We hypothesized that the slight transient facial nerve damage caused by the sponge contact suppressed facial nerve irritation, resulting in early postoperative spasm relief. Furthermore, the degree of preoperative compression of the nerve by the offending vessels may be associated with symptom improvement.

Approximately 7% of HFS cases develop postoperative recurrence after MVD [12], which is consistent with our results. Due to the use of several materials, the interposition method has been associated with long-term complications, such as prosthesis hardening, fibrosis, and chronic inflammation [13]. However, our study showed comparable outcomes and recurrence rates between the interposition (using a classic sponge) and transposition methods, although follow-up durations significantly varied significantly. These discrepancies may be attributed to the unique approach of our study, which included a single surgeon who performed both techniques in a standardized manner, as compared to previous studies involving multiple surgeons or diverse techniques.

The transposition method, despite its numerous variations [10, 18], is generally more time-consuming, technically challenging, and carries higher complication risks compared to the interposition method [7]. Therefore, interposition may be a viable alternative when transposition is deemed high-risk for select cases. Prior studies have documented recurrence associated with the use of diverse interposition materials between the compressing vessel and REZ [3, 11, 14]. Conversely, our study showed that comparable recurrence rates could be achieved when using only a sponge for the interposition method.

Despite useful insights offered in this study, several limitations should be acknowledged. First, the sample size was relatively small. Second, multivariate analysis for early spasm relief was not conducted. Third, the potential influence of surgeon experience was not considered in the study. Finally, the choice of the MVD methods in each patient was not randomized. Further studies with larger samples and longer follow-up periods are warranted to confirm our findings. Nevertheless, this study provides valuable information owing to its unique approach in employing a single surgeon using standardized techniques.

## Conclusions

This study investigated the outcomes of MVD treatment for HFS performed by a single surgeon using two standardized techniques. Our findings demonstrate that both the transposition (Surgicel® and fibrin glue) and interposition (sponge) methods are effective surgical approaches. Notably, the interposition method may achieve earlier resolution of the facial spasms.

## Data Availability

Data is available upon reasonable request.
